# Bleeding Symptoms in Pediatric Patients with Congenital FVII Deficiency and Correlation to Thrombin Generation Assay Parameters: A Single-Center Retrospective Analysis

**DOI:** 10.3390/life14121559

**Published:** 2024-11-27

**Authors:** Giovina Di Felice, Sonia Iavarone, Anna Lisa Montemari, Costanza Tripiciano, Michela Massoud, Simona Pezzi, Chiara Giorni, Ottavia Porzio, Matteo Luciani

**Affiliations:** 1 Clinical Laboratory Unit, Bambino Gesù Children’s Hospital, IRCCS, 00165 Rome, Italy; annalisa.montemari@opbg.net (A.L.M.); simona.pezzi@opbg.net (S.P.); ottavia.porzio@opbg.net (O.P.); 2 Onco-Hematology, Cell and Gene Therapy and Bone Marrow Transplant Clinic Area, Bambino Gesù Children’s Hospital, IRCCS, 00165 Rome, Italy; sonia.iavarone@opbg.net (S.I.); michela.massoud@opbg.net (M.M.); matteo.luciani@opbg.net (M.L.); 3 Infectious Disease Unit, Bambino Gesù Children’s Hospital, IRCCS, 00165 Rome, Italy; costanza.tripiciano@opbg.net; 4 Pediatric Cardiac Intensive Care Unit, Department of Cardiology and Cardiac Surgery, Bambino Gesù Children’s Hospital, IRCCS, 00165 Rome, Italy; chiara.giorni@opbg.net; 5 Department of Experimental Medicine, University of Rome Tor Vergata, 00133 Rome, Italy

**Keywords:** Factor VII, children, bleeding, thrombin generation

## Abstract

Inherited factor VII deficiency is the most common rare bleeding disorder, affecting about 1/500,000 individuals without gender predilection. Most of the patients with FVII 20–50% are asymptomatic, but post-traumatic or post-surgical bleeding may often occur since there is not an exact correlation between FVII plasma levels and the bleeding phenotype. We enrolled 19 children and adolescents with FVII levels of 20–35% and 33 controls. Laboratory data collected included thrombin generation, prothrombin time, activated partial thromboplastin time, fibrinogen, and FVII levels. In our study, we found a statistical difference in the lag time ratio (*p* < 0.01) and tt-peak ratio (*p* < 0.05) between patients and controls but no difference in the other parameters, such as the endogenous thrombin potential (ETP). However, when we categorized patients, regardless of their bleeding scores, as presenting symptoms and having no symptoms, both the lag time ratio (*p* = 0.01) and tt-peak ratio (*p* < 0.05) were significantly different, and the vel. index % showed increased levels in patients without symptoms (*p* < 0.05). This study shows that thrombin generation may be a useful tool in assessing the risk of bleeding symptoms in children with an FVII deficiency categorized in the mild category (20–35%), although we cannot predict the severity of the bleeding.

## 1. Introduction

Inherited factor VII deficiency (FVII D) is the most prevalent rare bleeding disorder (RBD), affecting approximately 1/500,000 people with no gender predisposition [1,2,3,4]. FVII D should be suspected if the prothrombin time (PT) is prolonged, where the activated partial thromboplastin time (aPTT) is normal. The PT value is subsequently adjusted in vitro using a mixing study with normal pooled plasma [5,6,7]. FVII D is an autosomal recessive disorder characterized by a number of genetic abnormalities, resulting in significant variations in both the genotype and phenotype [1,3,8,9], ranging from asymptomatic to severe, life-threatening bleeding.

The International Society of Thrombosis and Hemostasis categorizes patients based on FVII plasma levels into three categories of bleeding risk: (a) severe (risk of spontaneous bleeding < 10%), (b) moderate (risk of mild spontaneous or trigger bleeding for FVII 10–20%), and (c) mild (20–50%) [3,10].

The clinical picture may range from asymptomatic to potentially fatal bleeding events. The literature reports that levels of FVII < 2% may correlate with an increased risk of severe bleeding, but it may be possible that a lack of symptoms is possible in patients with an FVII level < 1% or severe bleeding in patients with greater levels of FVII [10,11]. The majority of people in the mild class, which has the highest prevalence, are asymptomatic [11] or may experience one or more episodes of light bleeding, such as mucocutaneous bleeding, epistaxis, easy bruising, or bleeding gums. In this group of patients, diagnosis can be unpredictable until a traumatic or surgical event occurs [8]. Individuals with certain levels of factor VII activity may experience massive hemorrhages, whereas others with the same levels are completely asymptomatic [1].

This disparity complicates illness management, particularly in pediatric settings [2]. Clinical symptoms are infrequent when FVII exceeds 30%. This value can thus be considered the clinical manifestation threshold [11], but protection from bleeding is not guaranteed. Even FVII levels above 20% may be protective against spontaneous bleeding but not against provoked bleeding, and, again, the phenotype may be variable. Detecting individuals at risk of post-surgical or gynecological bleeding is a clinical challenge [8]. Accordingly, identifying bleeding predictors may play a critical role in the therapeutic care of patients in the mild group [7], particularly those falling within the first half of that group with FVII plasma levels ranging from 20% to 35%. In fact, although bleeding phenotypes are not correlated to the factor levels, greater FVII plasma levels indicate a lower risk of bleeding.

Personal and family medical history appears to be the most reliable prediction strategy for determining bleeding risks [10]. Bleeding in the perinatal period and during the first year of life is common in infants with a more severe phenotype [10,12], although it may be absent in those with a mild condition. 

The Scientific Standardization Committee of the International Society of Thrombosis and Hemostasis (ISTH/SSC) has established a bleeding assessment tool (BAT) to assess the phenotype of hereditary bleeding diseases in children and adults, thereby facilitating clinical decision-making. The BAT is intended to reveal a history of mucosal bleeding, bleeding from minor wounds, systemic bleeding such as hematuria, melena, hematemesis, and bleeding following surgery or serious trauma, and a BAT ≥ 3 is predictive of a bleeding phenotype [10,13,14].

Accurate knowledge of a hemostatically safe status is critical in clinical practice, and FVII plasma levels and clinical history are currently the only instruments for predicting all forms of bleedings in all patients. 

The thrombin generation assay (TGA) is a valuable tool for investigating the coagulation process in all phases. TGA allows you to assess the quantity and rate of production of thrombin, which is an important molecule in the coagulation cascade. The assay determines the time needed to detect the thrombin formation onset (lag time), the quantity of thrombin formed within a certain time (ETP), the maximum quantity of generated thrombin (peak height), and the time needed to reach the peak height (time-to-peak).

The TGA assay has been widely utilized in research, and the availability of automated devices with standardized assays makes it feasible to use this tool in clinical practice. 

The purpose of this study was to identify bleeding phenotypes by comparing thrombin production assay parameters to bleeding symptoms in children in the first fraction of the mild group (20–35%).

## 2. Patients and Methods

This is a monocentric, observational, retrospective study on pediatric patients with an FVII deficiency, referred to the Pediatric Hematology and Oncology Department of Bambino Gesù Children’s Hospital in Rome in the period between January 2019 and July 2022. The study was conducted in accordance with the Declaration of Helsinki and approved by the Ethics Committee of the Bambino Gesù Children’s Hospital (Registration Number: 2989_OPBG_2022).

### 2.1. Subjects

Nineteen children and adolescents (2–17 years) with a congenital FVII deficiency with FVII plasma levels ranging from 20% to 35% were enrolled in the study after providing informed consent. Patients with an FVII above 35% and under 20% were excluded from the study. Clinical data were retrieved from medical records and gathered on a study database. Examination data and medical history were collected by the attending physician during routine visits, along with clinical data such as comorbidity, timing, severity and frequency of symptoms, bleeding score, treatment, family history, umbilical cord bleeding, dental bleeding, bruising, muscle hematomas or hemarthrosis, abnormal wound healing, epistaxis, and mucocutaneous bleeding. The assessment of the bleeding evaluation was based on the Bleeding Assessment Tool (BAT) proposed by the International Society on Thrombosis and Haemostasis (ISTH). Most of the patients showed no or just mild bleeding without reaching a positive bleeding score. We subdivided our group of patients into two sub-groups, the asymptomatic group (group A) and the symptomatic group (group S), without consideration of their bleeding scores.

The congenital nature of an FVII deficiency was established based on the family history. Acquired FVII deficiencies were excluded from the study, including a vitamin K deficiency and liver diseases.

Patients with a mental incapacity, poor cooperation, or language barriers that precluded an understanding of the purpose of the study and adequate cooperation were excluded from the study.

### 2.2. Controls (No FVII Deficiency)

Thirty-three individuals (2–17 years) who were referred to our hospital for minor issues other than hemostasis problems or a routine clinical check-up were enrolled as controls in the study after providing informed consent. Clinical data were retrieved from medical records, and subjects reporting a history of bleeding, hematological, liver, or cardio-vascular diseases, anticoagulant treatment, or acute/chronic infections within the previous thirty days were excluded from the study.

### 2.3. Laboratory Data Collection

We retrospectively collected laboratory data on patients and controls in the study database.

### 2.4. Bleeding Assessment Tool (BAT)

The patients’ ISTH/SSC BAT scores were reported in the study database in order to classify patients based on the bleeding phenotype. The scores had earlier been registered in the medical records by clinicians after a detailed survey of patients and their parents during routine visits. The BAT positive cut-off score was ≥3 [10,12].

### 2.5. Laboratory Determinations

Laboratory data collected included thrombin generation data (TG), the prothrombin time (PT), the activated partial thromboplastin time (aPTT), and fibrinogen and FVII levels at the diagnosis and on the day of the thrombin generation test. Laboratory tests were carried out by the laboratory at the “Bambino Gesù” Children’s Hospital (IRCCS) in Rome, Italy.

### 2.6. Thrombin Generation Assays

Thrombin generation assays are “global” assays that go beyond endpoint tests to measure the dynamics of the blood coagulation process. Thrombin generation parameters included the lag time, time-to-peak, peak thrombin generation (peak), endogenous thrombin potential (ETP), and thrombin generation velocity. Lag time is measured in minutes and a ratio. The peak height, also known as the peak thrombin, is expressed in molar concentrations of thrombin. The time to peak is expressed in minutes. The start tail is expressed in minutes and a ratio. The velocity index is expressed in nM/min and %. ETP is expressed in nM/min and %.

Samples were analyzed using ST GenesIIa (Diagnostica Stago, Asnières sur Seine, France), and data retrieved were added to the study database.

Calibration curves were created using the STG-Cal&Fluo kit, which included STG-ThrombiCal, STG-FluoStart, and STG-FluoSet. Following a successful calibration, we ran quality controls and reference plasma samples (STG-RefPlasma). The commercial GenesIIa STG-BleedScreen kit was used for investigations. Plasma samples from patients and controls were thawed in a warm bath at 37 °C for 1–2 min, gently mixed, and analyzed. Fluorescence was measured at wavelengths of 450 nm every 15 s for emissions and at 377 nm for absorption. The previously mentioned STG-RefPlasma process was run pa rallel to each sample. This reference plasma data, provided with assigned ranges on a barcoded flyer, assisted in normalizing results. The formula [Patient sample result/Reference plasma result * Activity allocated for the specific lot and parameter of this reference plasma] was used to calculate the normalized values for each sample. This normalization computation was carried out automatically by the ST GenesIIa instrument (Diagnostica Stago, Asnières sur Seine, France).

### 2.7. Routine Laboratory Measurements

#### PT, aPTT, Fibrinogen and FVII

The STA-NeoPTimal reagent (Diagnostica Stago, Asnières sur Seine, France) was used to measure the prothrombin time (PT). The clotting time in seconds (sec.) was used to represent PT values. The activated partial thromboplastin time (aPTT) was measured using the STA-Cephascreen reagent (Diagnostica Stago, Asnières sur Seine, France). The clotting time in seconds was used to report aPTT data.

The STA-Liquid Fib reagent (Diagnostica Stago, Asnières sur Seine, France) was used to measure fibrinogen, which was reported in mg/dL and measured using the Clauss method.

The clotting activity of FVII was measured through PT-based assays using the respective deficient substrate plasmas, expressed in %.

Samples were analyzed using a STAR Max^2^ coagulation analyzer (Diagnostica Stago, Asnières sur Seine, France).

All the measurements were made in accordance with the manufacturer’s protocol.

Our laboratory’s reference values were considered as defining normal ranges: PT 11.7–14.7 s, aPTT 26–39.6 s, fibrinogen 187–501 mg/dL, and FVII 50–140%.

### 2.8. Counts of Hemoglobin, White Blood Cells and Platelets Count

Blood was collected in K2E EDTA tubes (BD 3 mL), and the count was performed using the ADVIA 2120i analyzer (Siemens Healthcare Diagnostics S.r.l., Milano, Italy). All the measurements were made in accordance with the manufacturer’s protocol.

### 2.9. Statistical Analysis

Descriptive data were summarized as the median and interquartile range (continuous variables) or counts and percentages (categorical variables).

The normality of the distribution of continuous variables was checked using the Shapiro–Wilk test.

Comparisons between groups were performed using the Mann–Whitney U-test in the case of continuous variables and the chi-square test or Fisher’s exact test for categorical variables, as appropriate.

Correlations were assessed through the Spearman coefficient.

A *p* value < 0.05 was considered statistically significant.

Statistical analyses were performed using the IBM SPSS statistics 23.0 software.

## 3. Results

**Figure 1:**
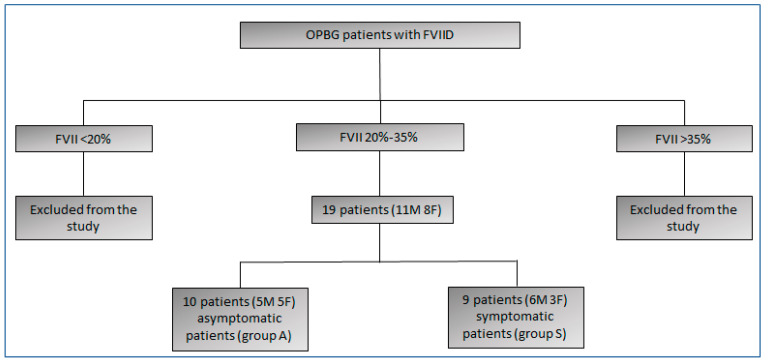
Flow diagram of patients.

We enrolled 19 patients (11 males and 8 females) (Figure 1) and 33 controls (15 males and 18 females) in the study. The patients’ mean age was 11.8 ± 3.4, and the mean age of the controls was 11.4 ± 4.6. Characteristics of enrolled patients are reported in Supplemental Materials Table S1.

Prothrombin time ranged from 17.5 s to 22 s (mean 19.0 ± 1.1), and the activated partial thromboplastin time from 23.2 to 33 s (mean 29.8 ± 2.4). Fibrinogen levels ranged from 210 mg/dL to 415 mg/dL (mean 276 ± 57), and the number of platelets was from 145 × 10^3^ µL to 596 × 10^3^ µL (mean 258 ± 99). The bleeding score and symptoms were found in clinical records. Ten patients presented a score of 0 and no symptoms, five patients a score of 1 (four patients with epistaxis, and one with post-trauma bruising), two patients a score of 3 (one patient with epistaxis, post-trauma bruising, and bleeding after tooth extraction, and one patient with postoperative bleeding treated with antifibrinolytics), and two patients a score of 4 (epistaxis, post-trauma bruising, post-trauma muscle hematoma and menorrhagia).

In Supplemental Materials Table S2, we have listed the results for all parameters of the thrombin generation tests noted on clinical records.

**Figure 2:**
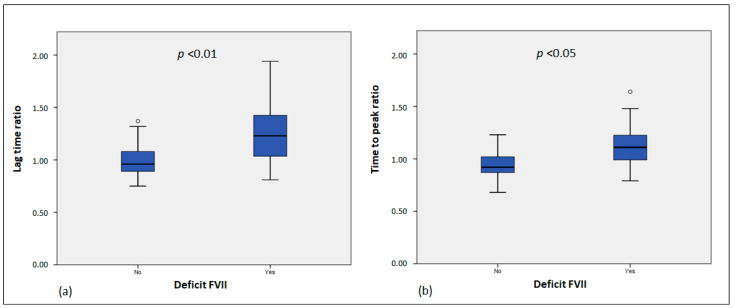
Lag time ratio and time-to-peak ratio in the patient group and control group. (**a**) Lag time ratio comparison between patients with FVII deficiency and controls; (**b**) Time-to-peak ratio comparison between patients with FVII deficiency and controls. Values that are more than 1.5 × IQR *below* Q1 or *above* Q3 are represented by circles (mild outliers). IQR = Interquartile range; Q1 = lower quartile; Q3 = upper quartile.

A comparison analysis of thrombin generation parameters in the patients with an FVII deficiency showed significant differences in the lag time (*p* = 0.02), lag time ratio (*p* = 0.003), and time-to-peak ratio (*p* = 0.04) compared to control subjects (Table 1; Figure 2).

**Figure 3:**
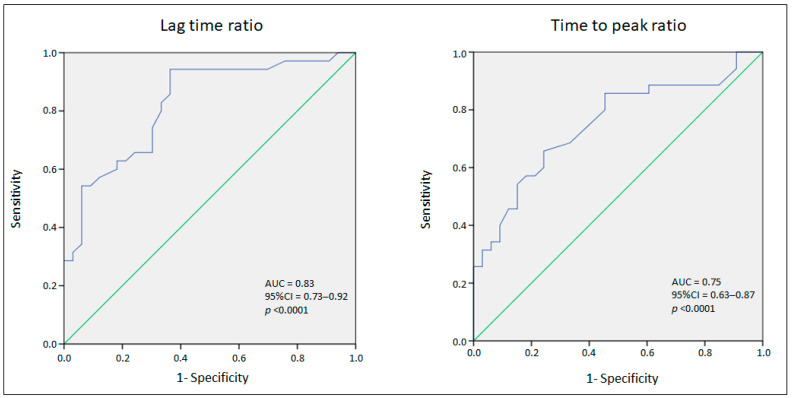
Thrombin generation and FVII deficiency ROC analysis.

The assessment of sensitivity and specificity of thrombin generation for the detection of an FVII deficiency was performed by an ROC analysis. The lag time ratio showed a good predictive ability in identifying a FVII deficiency, with an area under the ROC curve (AUC) of 0.83 (95% CI 0.73–0.92; *p* < 0.0001). The best cut-off, identified using the Youden index, was 0.975 (sensitivity 94.3%; specificity 63.6%). The time-to-peak ratio showed a fair predictive ability in identifying an FVII deficiency, with an area under the ROC curve (AUC) of 0.75 (95% CI 0.63–0.87; *p* < 0.0001). The best cut-off, identified using the Youden index, was 1.03 (sensitivity 65.7%; specificity 75.8%) (Figure 3).

Correlation analysis between patients FVII levels and thrombin generation results, PT and FVII levels, and PT and TGA results did not show significant results, and neither did a comparison of FVII levels between group A and group S (Table S3).

**Figure 4:**
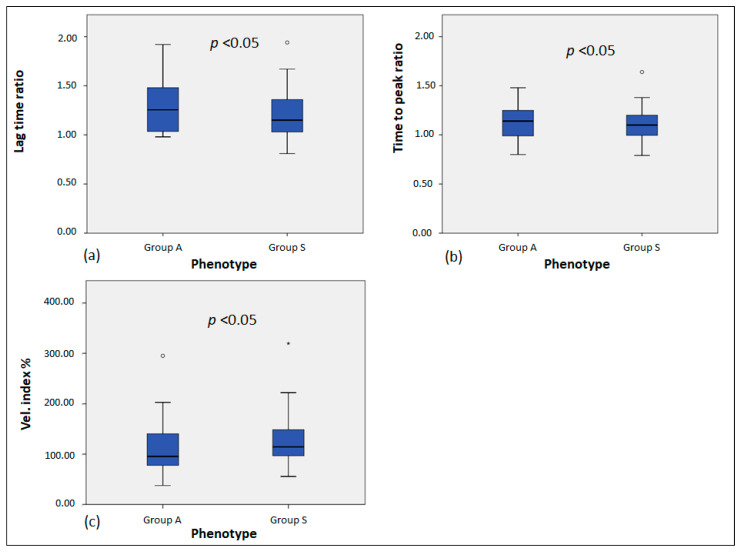
Lag time ratio, time-to-peak ratio, and vel. index % in the patient groups A and S (**a**) lag time ratio comparison between FVII deficient patients in group A and group S; (**b**) Time-to-peak ratio comparison between FVII deficient patients in group A and group S; (**c**) vel. index % ratio comparison between FVII deficient patients in group A and group S. Values that are more than 1.5 × IQR *below* Q1 or *above* Q3 are represented by circles (mild outliers). Values that are more than 3.0 × IQR *below* Q1 or *above* Q3 are represented by asterisks (extreme outliers). IQR = Interquartile range; Q1 = lower quartile; Q3 = upper quartile.

Analysis of thrombin generation results between groups A and S revealed that the time-to-peak (*p* = 0.03), lag time ratio (*p* = 0.01), time-to-peak ratio (*p* = 0.02), and vel. index% (*p* = 0.03) were significantly different between the two groups of patients (Table 2 and Figure 4).

**Figure 5:**
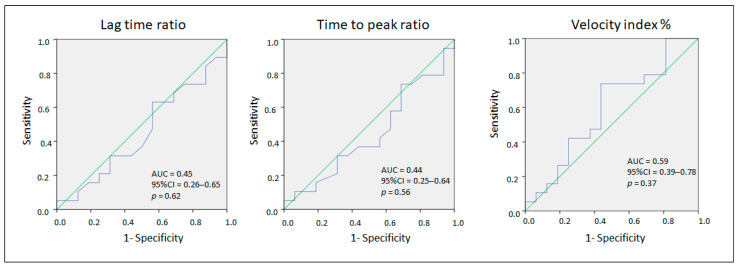
Thrombin generation and ROC analysis.

The ROC analysis for the lag time ratio, time-to-peak ratio, and velocity index% showed poor performance in identifying symptomatic individuals (Figure 5).

## 4. Discussion

The degree of FVII activity is not a reliable index of the risk and severity of bleeding symptoms. According to research and clinical experience, the bleeding phenotype and severity of an FVII deficiency are unrelated [5].

A critical issue in the routine management of pediatric patients with an FVII deficiency is the identification of subjects who need prophylaxis or replacement therapy in the event of a high risk of bleeding, such as surgery or trauma [15]. Prophylaxis should be prescribed in childhood soon after the first bleeding event [16].

Replacement therapy with an FVII plasma-derived concentrate or recombinant activated factor VII (rFVIIa) is the main treatment, but dosage schedules have not yet been determined in pediatric populations [17], despite the fact that safety and effectiveness have been assessed in hemophiliac patients [18]. Investigations have shown that very modest dosages are adequate for treatment [19], and prophylaxis [20] and long-term prophylaxis with dosages of 10–30 U/kg (pdFVII) or higher have been described by Siboni et al. However, the dosages were adjusted based on a clinical follow-up [21], and clinical experience is actually the key to FVII deficiency management in childhood [22]. Alongside replacement therapy, antifibrinolytic medications such as tranexamic acid are an option in the management of symptoms, especially for mucocutaneous bleedings, menorrhagia, or minor surgery, such as dental surgery [8,23].

Bleeding score tools, such as a Mariani score or the Di Minno patients’ classification [2,22], are currently used to assess the probability of bleeding in patients with an FVII deficiency, but there is no correlation between the factor VII activity level and bleeding score, despite the fact that the bleeding score is a valuable clinical tool for assessing patients with an elevated bleeding risk [4,24,25].

Several investigations have tried to use clinical and laboratory techniques to predict the hemorrhagic phenotype of individuals with a factor VII deficiency, but the findings have been inconsistent [5]. TGAs have reportedly been successful in predicting the clinical phenotype of RBDs and hemophilia, according to certain research [6,26]. It is debatable, though, whether the bleeding phenotype of factor VII deficient patients can be predicted using a global assay [27,28].

In 27 FVII-deficient patients, Toret et al. compared global assays, thromboelastography (TEG), and thrombin generation (TGAs) to the bleeding score. These authors found that the BS is a more relevant measure and that both tests are unable to predict the bleeding phenotype [5,29]. According to Pasca et al., TGA may be used to distinguish between patients with severe factor VII deficiency and those with moderate or mild deficiency but not between the two latter categories [29]. The Mariani score, the Di Minno classification, and the ISTH/SCC-BAT score are the three bleeding scores that were compared to the TGA parameters. For factor VII lag time and tt-peak plasma levels, there was a statistically significant (*p* < 0.05) difference when compared to a healthy control group and a varied lag time when comparing different phenotypes.

In our study, as for those authors, we discovered a statistically significant difference in the lag time ratio (*p* < 0.01) and tt-peak ratio (*p* < 0.05) between patients and controls, but this difference was not apparent for the other metrics, such as the ETP. However, there were significant differences in the lag time ratio (*p* = 0.01), tt-peak ratio (*p* < 0.05), and vel. index% (*p* < 0.05) when patients were categorized based on whether they had symptoms (group S) or not (group A). This study suggests that although the degree of the bleeding cannot be predicted, the TGA assay may be a useful tool in mild factor VII deficiencies in differentiating symptomatic and asymptomatic phenotypes. Our study may offer a new technique for identifying those in the mild group of individuals with a factor VII deficiency that may have a symptomatic phenotype, and it also partially validates what is already known in the literature. The small sample size and the subjects’ young ages, who had age-limited exposure to potentially bleeding events, are undoubtedly major limitations of our study. Nevertheless, the homogeneity of the participants is a strength of our research, and it might be possible to recommend using the TGA assay in conjunction with BS as a useful method for identifying mild patients who are at risk of bleeding symptoms. However, more trials with a larger patient population and multicenter investigations are required.

## 5. Conclusions

According to this study, TGA may be a helpful method for determining the phenotype of bleeding symptoms in children with an FVII deficiency who fall into the milder category (20–35%). Multicenter investigations are required to corroborate the results, and traditional inquiries based on personal and family history of bleeding diathesis must always be carried out in all cases.

Further pediatric research is currently required, but a combination of medical history data, traditional laboratory testing, and thrombin generation data may one day make it possible to define the phenotypes of pediatric patients and ensure appropriate care, particularly for more ambiguous patients such as those in the lower range of the mild category FVII deficiency (20–35%).

## Figures and Tables

**Table 1:** Absolute and normalized mean values for the parameters of the thrombin generation test in relation to the presence of FVII deficiency.

	Parameter	FVII Deficiency Yes	FVII Deficiency No (Controls)	*p* *
	N	19	33	
**Absolute TGA parameters values**	**Lag time (min)**	3.15 [2.63–3.77]	2.52 [2.35–2.80]	**0.02**
	**Peak height (nM)**	142.4 [117.6–195.0]	173.3 [134.2–206.5]	0.51
	**Time-to-peak (min)**	6.06 [5.30–6.58]	5.11 [4.85–5.83]	0.054
	**ETP (nM/min)**	913 [807–1151]	1014 [850–1123]	0.66
	**Vel. index (nM/min)**	69.4 [46.7–97.1]	83.9 [58.3–116.2]	0.48
	**Start tail (min)**	19.7 [17.5–21.2]	17.8 [15.4–20.6]	0.27
**Normalized TGA parameters values**	**Lag time ratio**	1.23 [1.03–1.47]	0.96 [0.88–1.08]	**0.003**
	**Peak height %**	103.6 [87.0–129.1]	105.9 [86.4–143.3]	0.80
	**Time-to-peak ratio**	1.11 [0.98–1.25]	0.92 [0.87–1.05]	**0.04**
	**ETP%**	91.2 [78.1–106.4]	86.0 [77.6–98.2]	0.83
	**Vel. index %**	106.2 [84.5–148.8]	123.0 [84.9–159.8]	0.70
	**Start tail ratio**	0.89 [0.380–0.98]	0.83 [0.69–0.96]	0.25

* Mann–Whitney U-test.

**Table 2:** Absolute and normalized mean values of TGA parameters in group A vs. group S.

	Parameter	Group A	Group S	*p*
	N	10	9	
**Absolute TGA parameters values**	**Lag time**	2.76 [2.24–3.70]	3.29 [2.66–3.82]	0.21
	**Peak height (nM)**	162.9 [125.7–253.4]	159.0 [101.7–193.0]	0.16
	**Time-to-peak**	5.37 [4.53–6.53]	6.14 [5.55–7.05]	**0.03**
	**ETP (nM/min)**	959 [815–1295]	890 [727–1157]	0.11
	**Vel. index (nM/min)**	89.3 [37.4–141.1]	73.9 [47.2–88.5]	0.16
	**Start tail (min)**	18.6 [15.7–21.12]	18.5 [17.8–22.9]	0.32
**Normalized TGA parameters values**	**Lag time ratio**	1.08 [0.97–1.33]	1.31 [1.07–1.65]	**0.01**
	**Peak height %**	121.0 [86.5–156.9]	95.5 [66.1–123.3]	0.07
	**Time-to-peak ratio**	1.02 [0.85–1.29]	1.14 [1.02–1.29]	**0.02**
	**ETP%**	97.1 [80.4–110.3]	82.9 [67.8–99.3]	0.08
	**Vel. index %**	139.9 [100.1–186.5]	95.0 [66.2–131.5]	**0.03**
	**Start tail ratio**	0.86 [0.72–0.97]	0.86 [0.82–1.07]	0.16

## Data Availability

Clinical data are available from the last author upon reasonable request in accordance with the General Data Protection Regulation (EU) 2016/679.

## Supplementary Materials

The following supporting information can be downloaded at https://www.mdpi.com/article/10.3390/life14121559/s1, Table S1a. Patient characteristics; Table S1b. Laboratory data of total patients, group A patients, group S patients, and controls; Table S2. Absolute and normalized thrombin generation; Table S3. Mean values of FVII in relation to symptoms.

## References

[1] Mariani G., Herrmann F.H., Dolce A., Batorova A., Etro D., Peyvandi F., Wulff K., Schved J.F., Auerswald G., Ingerslev J. (2005). Clinical phenotypes and factor VII genotype in congenital factor VII deficiency. Thromb. Haemost..

[2] Di Minno M.N., Dolce A., Mariani G. (2013). Bleeding symptoms at disease presentation and prediction of ensuing bleeding in inherited FVII deficiency. Thromb. Haemost..

[3] Jain S., Donkin J., Frey M.J., Peltier S., Gunawardena S., Cooper D.L. (2018). Phenotypical variability in congenital FVII deficiency follows the ISTH-SSC severity classification guidelines: A review with illustrative examples from the clinic. J. Blood Med..

[4] Robinson K.S. (2019). An overview of inherited factor VII deficiency. Transfus. Apher. Sci..

[5] Toret E., Ay Y., Karapinar T.H., Oymak Y., Kavakli K., Vergin R.C. (2020). Evaluation of Bleeding Phenotype of Inherited Factor VII Deficiency in Children With a Bleeding Assessment Tool and Global Assays. J. Pediatr. Hematol./Oncol..

[6] Greene L.A., Goldenberg N.A., Simpson M.L., Villalobos-Menuey E., Bombardier C., Acharya S.S., Santiago-Borrero P.J., Cambara A., DiMichele D.M. (2013). Use of global assays to understand clinical phenotype in congenital factor VII deficiency. Haemophilia.

[7] Iwaniec T., Zdziarska J., Musiał J., Sanak M. (2019). A Retrospective Analysis of Clinical and Laboratory Data of Patients with Factor VII Deficiency: A Single Centre Experience. Hamostaseologie.

[8] Napolitano M., Siragusa S., Mariani G. (2017). Factor VII Deficiency: Clinical Phenotype, Genotype and Therapy. J. Clin. Med..

[9] Halimeh S., Koch L., Kenet G., Kuta P., Rahmfeld T., Stoll M., Nowak-Göttl U. (2023). Genotype-Phenotype Relationship among 785 Unrelated White Women with Inherited Congenital Factor VII Deficiency: A Three-Center Database Study. J. Clin. Med..

[10] Peyvandi F., Palla R., Menegatti M., Siboni S.M., Halimeh S., Faeser B., Pergantou H., Platokouki H., Giangrande P., Peerlinck K. (2012). Coagulation factor activity and clinical bleeding severity in rare bleeding disorders: Results from the European Network of Rare Bleeding Disorders. J. Thromb. Haemost..

[11] Sevenet P.O., Kaczor D.A., Depasse F. (2017). Factor VII Deficiency: From Basics to Clinical Laboratory Diagnosis and Patient Management. Clin. Appl. Thromb./Hemost..

[12] Lapecorella M., Mariani G. (2008). Factor VII deficiency: Defining the clinical picture and optimizing therapeutic options. Haemophilia.

[13] Rodeghiero F., Tosetto A., Abshire T., Arnold D.M., Coller B., James P., Neunert C., Lillicrap D. (2010). ISTH/SSC bleeding assessment tool: A standardized questionnaire and a proposal for a new bleeding score for inherited bleeding disorders. J. Thromb. Haemost..

[14] Elbatarny M., Mollah S., Grabell J., Bae S., Deforest M., Tuttle A., Hopman W., Clark D.S., Mauer A.C., Bowman M. (2014). Normal range of bleeding scores for the ISTH-BAT: Adult and pediatric data from the merging project. Haemophilia.

[15] Peltier S., Kellum A., Brewer J., Duncan A., Cooper D.L., Saad H. (2020). Psychosocial Impact and Disease Management in Patients with Congenital Factor VII Deficiency. J. Blood Med..

[16] Bernardi F., Mariani G. (2021). Biochemical, molecular and clinical aspects of coagulation factor VII and its role in hemostasis and thrombosis. Haematologica.

[17] Kuperman A.A., Barg A.A., Fruchtman Y., Shaoul E., Rosenberg N., Kenet G., Livnat T. (2017). Primary prophylaxis for children with severe congenital factor VII deficiency—Clinical and laboratory assessment. Blood Cells Mol. Dis..

[18] Konkle B.A., Ebbesen L.S., Erhardtsen E., Bianco R.P., Lissitchkov T., Rusen L., Serban M.A. (2007). Randomized, prospective clinical trial of recombinant factor VIIa for secondary prophylaxis in hemophilia patients with inhibitors. J. Thromb. Haemost..

[19] Brummel Ziedins K., Rivard G.E., Pouliot R.L., Butenas S., Gissel M., Parhami-Seren B., Mann K.G. (2004). Factor VIIa replacement therapy in factor VII deficiency. J. Thromb. Haemost..

[20] van Geffen M., Mathijssen N.C., Holme P.A., Laros-van Gorkom B.A., van Kraaij M.G., Masereeuw R., Peyvandi F., van Heerde W.L. (2013). Pharmacodynamics of recombinant activated factor VII and plasma-derived factor VII in a cohort of severe FVII deficient patients. Thromb. Res..

[21] Siboni S.M., Biguzzi E., Mistretta C., Garagiola I., Peyvandi F. (2015). Long-term prophylaxis in severe factor VII deficiency. Haemophilia.

[22] Strauss E.R., Mazzeffi M.A., Williams B., Key N.S., Tanaka K.A. (2017). Perioperative management of rare coagulation factor deficiency states in cardiac surgery. Br. J. Anaesth..

[23] Kim S.H., Park Y.S., Kwon K.H., Lee J.H., Kim K.C., Yoo M.C. (2012). Surgery in patients with congenital factor VII deficiency: A single center experience. Korean J. Hematol..

[24] Qu C., Liu W., Chen L., Zhang L., Xue F., Yang R. (2023). Factor VII deficiency in China: Phenotype, genotype and current status of management. Br. J. Haematol..

[25] Tripathi P., Mishra P., Ranjan R., Tyagi S., Seth T., Saxena R. (2019). Factor VII deficiency—An enigma; clinicohematological profile in 12 cases. Hematology.

[26] Yang Y., Zeng Y.C., Rumende P., Wang C.G., Chen Y. (2021). Diagnosis and treatment discussion of congenital factor VII deficiency in pregnancy: A case report. World J. Clin. Cases.

[27] Tran H.T., Tjønnfjord G.E., Holme P.A. (2014). Use of thromboelastography and thrombin generation assay to predict clinical phenotype in patients with severe FVII deficiency. Haemophilia.

[28] Valke L., Rijpma S., Meijer D., Schols S.E.M., van Heerde W.L. (2022). Thrombin generation assays to personalize treatment in bleeding and thrombotic diseases. Front. Cardiovasc. Med..

[29] Pasca S., Santoro C., Ambaglio C., Napolitano M., Milan M., Natali L., Nannizzi S., Mori F., Simioni P., Zanon E. (2022). Comparison among three different bleeding scores and the thrombin generation assay to assess the different hemorrhagic phenotypes in patients with FVII deficiency. Bleeding Thromb. Vasc. Biol..

