# Supplementary material for: Bleeding Symptoms in Pediatric Patients with Congenital FVII Deficiency and Correlation to Thrombin Generation Assay Parameters: A Single-Center Retrospective Analysis
Source: Life (Basel). 2024 Nov 27;14(12):1559. doi: 10.3390/life14121559 (PMC11678817; doi:10.3390/life14121559)
Supplement: Supplementary file 1 [file life-14-01559-s001.zip › life-3297908-supplementary.pdf]

Table S1a. Patient characteristics (GERD = gastroesophageal reflux disease; DIV= interventricular defect).

Patient	Age	Sex	FVII %	Bleeding Score	Comorbidities
**1**	17	M	27	1	-
**2**	14	M	25	0	-
**3**	8	M	33	0	-
**4**	6	F	24	0	-
**5**	12	F	24	1	-
**6**	8	F	34	0	cerebellar peduncle lesion
**7**	10	M	35	0	retractile testicle
**8**	10	M	30	1	-
**9**	17	M	23	0	-
**10**	14	F	32	0	-
**11**	12	F	28	4	GERD, migraine
**12**	12	M	25	0	-
**13**	6	M	35	1	pulmonary atresia with DIV
**14**	14	F	27	0	acrocyanosis
**15**	8	F	32	0	Klippel Trénaunay
**16**	13	M	22	1	-
**17**	11	M	35	3	-
**18**	14	F	34	4	endometriosis
**19**	14	M	35	3	esophageal atresia, bronchiectasis

Table S1b. Laboratory data of total patients, group A patients, group S patients, and controls (PT= prothrombin time; aPTT= activated partial thromboplastin time; Hb= hemoglobin (g/dl); Plts= platelets (10^3/mm^3); WBC= white blood cells (10^3/mm^3).

	Controls	Patients	p value	Group A	Group S	p value
**PT**	14.74±0.92	19.0±1.1	0.01	19.2±1.0	18.8±1.3	0.24
**aPTT**	28.29±2.33	29.8±2.4	0.057	29.4±2.69	30.3±1.95	0.50
**Fibrinogen**	291±48	276±57	0.43	275±54	275±63	0.9
**Hb**	13.06±1.51	14.00±2.06	0.18	14.33±0.58	13.67±3.15	0.95
**Plts**	256±62	258±99	0.49	259±48	255±138	0.91
**WBC**	7.67±2.82	6.29±2.16	0.147	5092±1713.90	6618±2636.30	0.11

Table S2. Absolute andnormalized thrombin generation (Pt.= patient; LT=lag time; PH= peak heigh; TtP= time to peak; ETP= endogenous thrombin potential Vel=vel. index; ST=start tail; LTr=lag time ratio; TtPr= time to peak ratio; STr=start tail ratio).

	**Absolute TGA parameters values**						**Normalized TGA parametes values**				
**Pt.**	**LT (min)**	**PH (nM)**	**TtP (min)**	**ETP (nM/min)**	**Vel (nM/min)**	**ST (min)**	**LTr**	**PH%**	**TtP r**	**ETP%**	**ST r**
**1**	2.55	139.5	5.36	980.2	75.53	20.96	0.99	96.04	0.98	91.24	0.96
**2**	4.58	239.2	6.87	1206	134.0	17.93	1.78	164.7	1.25	112.3	0.82
**3**	4.94	76.78	8.14	700.3	31.65	30.33	1.92	52.87	1.48	65.19	1.39
**4**	3.37	126.7	6.06	753.6	62.73	18.53	1.31	87.26	1.11	70.5	0.85
**5**	2.61	367.9	4.59	1786	249.1	16.11	1.02	220.4	0.85	152.1	0.77
**6**	3.77	215.5	6.73	1223	89.91	17.95	1.47	129.1	1.25	104.2	0.86
**7**	2.63	132.5	5.07	841.1	73.86	19.69	1.03	79.37	0.94	71.63	0.94
**8**	2.57	209.2	5.44	1302	94.25	18.61	1	125.4	1.01	110.9	0.89
**9**	2.8	159.3	6.14	1108	65.89	20.42	1.09	95.46	1.14	94.4	0.98
**10**	2.69	170.5	5.35	913.2	87.15	16	1.04	117.4	0.98	85.01	0.73
**11**	3.88	84.3	6.9	580.2	37.64	21.47	1.51	58.05	1.26	54.01	0.98
**12**	3.87	59.19	7.23	498.8	24.85	25.43	1.51	40.76	1.32	46.43	1.16
**13**	2.28	176.3	4.65	937.1	97.08	16.25	0.89	121.4	0.85	87.23	0.74
**14**	3.29	159	5.74	890.5	86.49	17.72	1.28	109.5	1.05	82.9	0.81
**15**	3.83	91.69	7.33	701.6	36.56	22.83	1.49	54.94	1.36	59.75	1.09
**16**	2.9	257.8	5.3	1293	136.1	16.25	1.13	154.4	1.38	110.1	1.16
**17**	3.51	149.4	5.88	852.8	84.36	18.57	1.23	120.5	1.02	93.1	0.83
**18**	2.11	251.9	4.33	1199	155.9	14.4	0.81	164.5	0.79	108.9	0.67
**19**	3.65	137	6.41	925.3	74.73	20.36	1.28	110.5	1.11	101	0.91

Table S3. Mean values of FVII in relation to symptoms.

	Group A	Group S	P*
**N**	10	9	
**FVII**	31 [26.3-35]	27 [24.5-33.5]	0.55

* Mann–Whitney U-test
